# Binarization of enhanced depth imaging optical coherence tomographic images of an eye with Wyburn-Mason syndrome: a case report

**DOI:** 10.1186/s12886-015-0014-2

**Published:** 2015-03-07

**Authors:** Akiko Iwata, Yoshinori Mitamura, Masanori Niki, Kentaro Semba, Mariko Egawa, Takashi Katome, Shozo Sonoda, Taiji Sakamoto

**Affiliations:** Department of Ophthalmology, Institute of Health Biosciences, The University of Tokushima Graduate School, 3-18-15, Kuramoto-cho, Tokushima, 770-8503 Japan; Department of Ophthalmology, Kagoshima University Graduate School of Medical and Dental Sciences, 8-35-1 Sakuragaoka, Kagoshima, 890-8520 Japan

**Keywords:** Binarization, Choroidal structure, Enhanced depth imaging optical coherence tomography, Fluorescein angiography, Wyburn-Mason syndrome

## Abstract

**Background:**

To report a thicker choroid and larger choroidal luminal area in an eye with Wyburn-Mason syndrome. To the best of our knowledge, this is the first report demonstrating an increase in the choroidal thickness and the luminal area in a case of Wyburn-Mason syndrome. In addition, we report the changing appearance of retinal arteriovenous malformations over a 16-year period.

**Case presentation:**

A 27-year-old woman, who was diagnosed with Wyburn-Mason syndrome at age 11 years, visited our clinic. Her best-corrected visual acuity was 20/12.5 in the right eye and light perception in the left eye. Severely dilated, tortuous vascular loops were distributed from the optic disc over all four quadrants of the left fundus. The vascular loops in some areas were more dilated and tortuous than 16 years earlier. Optical coherence tomography (OCT) showed retinal edema with cystic changes and enlarged choroidal vessel lumens in the left eye. The subfoveal choroidal thickness was manually measured by the caliper function in the enhanced depth imaging OCT (EDI-OCT) images. Binarization of the EDI-OCT images was performed with publicly accessible ImageJ software. The examined area of the subfoveal choroid was 1,500 μm wide, and the dark areas representing the luminal areas were traced by the Niblack method. After determining the distance of each pixel, the luminal area was automatically calculated. The subfoveal choroidal thickness was 250 μm in the right eye and 462 μm in the left eye. The luminal area of the 1,500-μm-wide subfoveal choroid was computed to be 307,165.6 μm^2^ in the right eye and 545,780.7 μm^2^ in the left eye.

**Conclusions:**

The EDI-OCT images showed a thicker choroid, and binarization of the EDI-OCT images showed that the luminal areas were significantly larger in the affected eye, suggesting a dilatation of the choroidal vessels. The results demonstrated that conversion of EDI-OCT images to binary images was a useful method to quantify the choroidal structure.

## Background

Wyburn-Mason syndrome is a rare nonhereditary congenital disease characterized by arteriovenous malformations (AVMs) involving the brain, retina, and orbit. Spectral-domain optical coherence tomography (SD-OCT) of the eye with Wyburn-Mason syndrome showed a pattern of circular lesions corresponding to cross sections of the abnormal retinal vessels [1]. To date, there has not been a study of choroidal structure in eyes with Wyburn-Mason syndrome by enhanced depth imaging OCT (EDI-OCT). We recently reported that EDI-OCT images can be converted to binary images, which can then be used to quantify the luminal and interstitial areas of the choroid [2]. We applied this technique to quantify the luminal and stromal areas of the choroid in an eye with Wyburn-Mason syndrome. Approval was obtained from the Institutional Review Board of Tokushima University Hospital to perform this study, and the patient gave her written informed consent prior to her inclusion in the study.

## Case presentation

**Figure 1:**
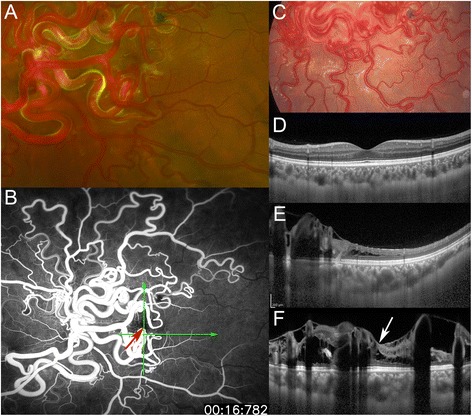
**Fundus photographs** **,** **fluorescein angiographic** **(FA)** **image,** **and enhanced depth imaging optical coherence tomographic** **(EDI-** **OCT)** **images. A**: Fundus photograph at age 27 years. Markedly dilated tortuous vascular loops consistent with retinal arteriovenous malformations are distributed from the optic disc over four quadrants in the left eye. Arteries are directly connected to veins without passing through any capillaries. The optic disc is obscured by very large vascular loops. Numerous anastomosing vessels make it difficult to separate the arterial and venous components. **B**: At age 27 years, wide-field fluorescein angiography in early phase shows fluorescein throughout the vascular loops without an intervening capillary bed and leakage from the loops, indicating a direct arteriovenous communication. Red arrow indicates the fovea (center of the foveal avascular zone), and green arrows indicate the direction of the OCT scans in ‘**E**’ and ‘**F**’. **C**: Fundus photograph at age 11 years. The vascular loops in some areas are less dilated and tortuous than at 27 years in the left eye (see ‘A’). **D**-**F**: EDI-OCT images in the healthy right eye **(D)** and the affected left eye **(E,** **F)** at age 27 years. Choroidal thickness of the left eye is thicker than that of the fellow eye. ‘**E**’ indicates a horizontal scan, and ‘**D**’ and ‘**F**’ indicate vertical scans through the fovea. OCT images in the left eye **(E,** **F)** demonstrate retinal edema with cystic changes and oval-shaped lesions corresponding to cross sections of abnormal retinal vessels. White arrow indicates cystoid macular edema **(F)**.

A 27-year-old woman, who was diagnosed with Wyburn-Mason syndrome at age 11 years, visited our clinic for a regular examination. At age 11 years, her best-corrected visual acuity (BCVA) was 20/500 in the left eye, and cerebral angiography demonstrated AVMs along the course of the left ophthalmic artery. There was no history of systemic diseases. On a regular examination at age 27 years, she was in the 8^th^ month of pregnancy, and her obstetrician consulted us on the risk of hemorrhaging from the retinal AVMs at the time of parturition. Her BCVA was 20/12.5 in the right eye and light perception in the left eye. Exophthalmos was not found, and the ocular motility was normal. The intraocular pressures were 16 mmHg OD and 17 mmHg OS. The anterior segments and ocular media of both eyes and fundus of the right eye were within normal limits. However, severely dilated tortuous vascular loops consistent with retinal AVMs were distributed from the optic disc over all four quadrants of the left eye (Figure 1A). The arteries were directly connected to the veins without passing through any capillaries. The optic disc was obscured by the large vascular loops. Numerous anastomosing vessels made it difficult to separate the arterial and venous components. The vascular loops in some areas were more dilated and tortuous than 16 years earlier (Figure 1C). Magnetic resonance imaging revealed orbital and retro-orbital AVMs including canalicular segments of the optic nerve on the left side. Prominent serpiginous vessels could be detected in the left orbit, including the apex which encased the optic nerve. Neurologic examinations were unremarkable, and no changes were found in the facial skin.

After delivering by Cesarean section, fluorescein angiography (FA) and indocyanine green angiography (IA) were performed. FA in the early phase showed a rapid transit of dye through the vascular loops without an intervening capillary bed and leakage from the loops, which indicated direct arteriovenous communications (Figure 1B). IA in the early phase (approximately 16 s after the injection of dye) also showed a transit of dye through the vascular loops. IA did not show any findings suggestive of choroidal AVMs, because details of the choroidal vessels in the posterior pole were obscured by the large retinal vascular loops.

**Figure 2:**
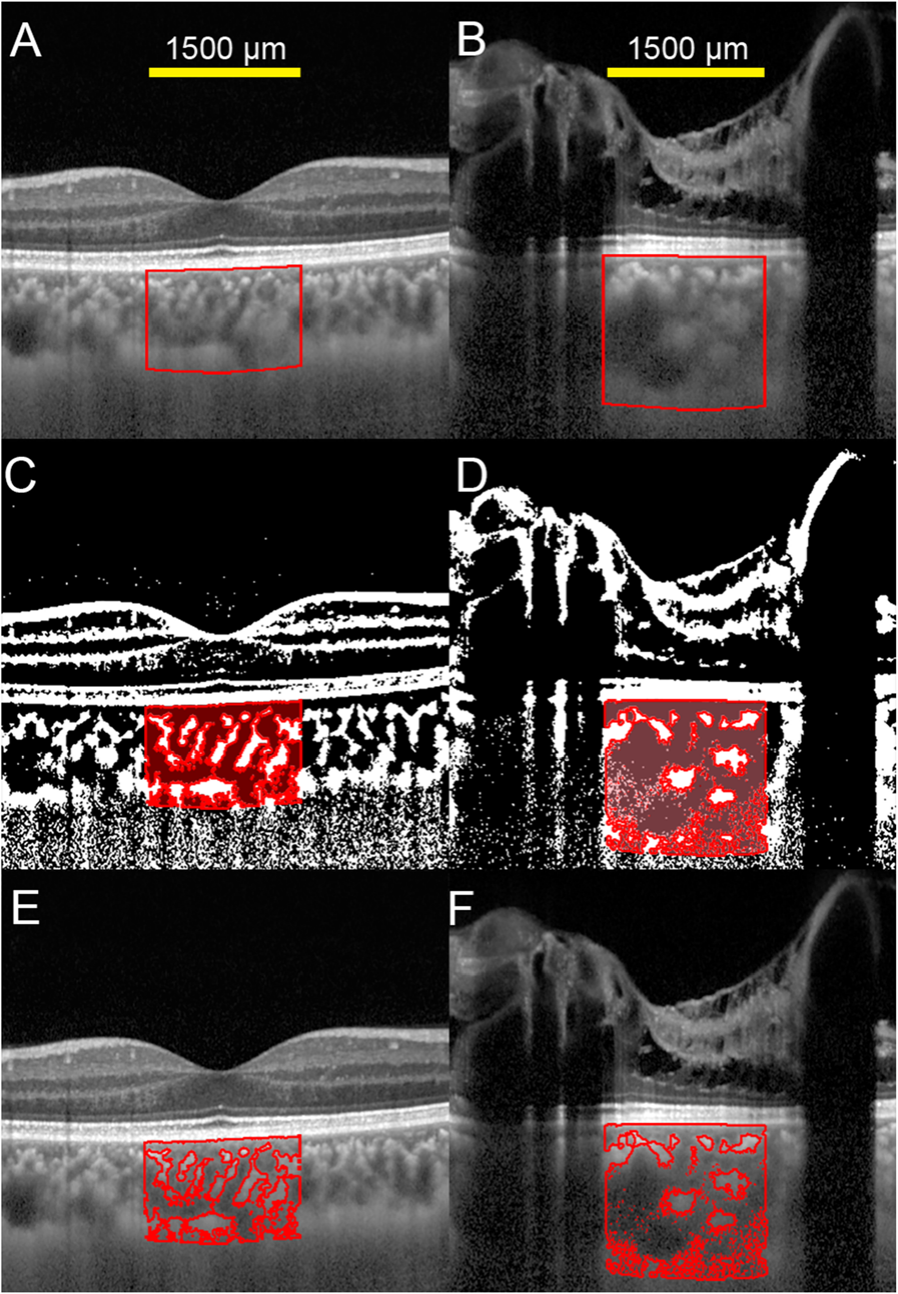
**Enhanced depth imaging optical coherence tomographic** **(EDI** **-OCT)** **images and converted binary images.** Left column shows EDI-OCT images of the right eye, and right column shows those of the left eye. EDI-OCT images of a vertical scan through the fovea **(A** **, B)** were converted to binary images using ImageJ software. **A**, **B**: The luminal area (dark area) and the interstitial area (light area) can be seen. The lumens of the choroidal vessels seem larger in the left eye than in the right eye. The examined area was determined to be 1,500 μm wide in the subfoveal choroid. It extended vertically from the retinal pigment epithelium to the chorioscleral border, and the choroidal area was set with the ROI manager of ImageJ. The rectangle surrounded by a red line was excised, and the dark areas were traced by the Niblack method. **C**, **D**: Merged images of the binarized images and the margins of traced areas. In the binarized images, the light pixels were defined as the interstitial choroid or choroidal stroma, and the dark pixels were defined as the luminal area. **E**, **F**: Merged images of the original EDI-OCT images and the margins of traced areas show that the traced areas coincide with the dark choroidal areas of the EDI-OCT image.

SD-OCT was performed with the Heidelberg Spectralis (Heidelberg Engineering, Heidelberg, Germany). SD-OCT demonstrated retinal edema with cystic changes and oval-shaped structures that represented cross sections of abnormal retinal vessels in the left eye (Figures 1E and 1 F). Binarization of a choroidal area in the EDI-OCT image was performed by a modified Niblack’s method as reported in detail (Figure 2) [2]. Briefly, an EDI-OCT image was analyzed by ImageJ software (ImageJ version 1.47, NIH, Bethesda, MD, USA). The examined area was 1,500 μm wide in the subfoveal choroid, and extended vertically from the retinal pigment epithelium to the chorioscleral border. This choroidal area was selected with the ImageJ ROI Manager. Three choroidal vessels with lumens larger than 100 μm were randomly selected by the Oval Selection Tool on the ImageJ tool bar, and the average reflectivity of these areas was determined. The average brightness was set as the minimum value to minimize noise in the OCT image. Then, the image was converted to 8 bits and adjusted by the Niblack Auto Local Threshold. The binarized image was converted to the RGB image again, and the luminal area was determined using the Threshold Tool. The light pixels were defined as the interstitial areas, and the dark pixels were defined as the luminal areas. After adding the data of the distance of each pixel, the luminal and interstitial areas were automatically calculated.

In the EDI-OCT images, the subfoveal choroidal thickness was computed to be 250 μm OD and 462 μm OS using the caliper function (Figures 1D and 1 F). The lumens of choroidal vessels appeared larger in the left eye (Figures 2A and 2B). In the binarized images, the luminal areas of the 1,500 μm wide choroid were 307,165.6 μm^2^ OD and 545,780.7 μm^2^ OS, while the interstitial areas were 175,593.5 μm^2^ OD and 273,833.0 μm^2^ OS. The percentages of the luminal areas relative to the total choroidal areas were 63.6% OD and 66.6% OS. Enlargement of the luminal area in the left eye was evident with accompanying interstitial enlargement.

## Discussion

Wyburn-Mason syndrome results from a defect occurring during embryonic development of the vascular mesoderm, related to the visual pathways passing from the retina to the mesencephalon [3]. The AVMs in the Wyburn-Mason syndrome result from the persistence of the embryonic vascular pattern that existed before the arteries, capillaries, and veins differentiated during the 2nd month of gestation [4].

The extent of the lesions varies widely, because the time of the insult to the embryonic tissue determines which structures are affected. Because our case had AVMs in the retina, orbit, and retro-orbital brain, there may have also been choroidal AVMs.

To the best of our knowledge, this is the first report showing a thicker choroid in an eye with Wyburn-Mason syndrome. The cause of the thickened choroid was not determined conclusively; however, there is a possibility that the presence of choroidal AVMs could have led to the increased choroidal thickness. In the EDI-OCT images, the lumens of the choroidal vessels appeared enlarged, which was confirmed by analyses of the binarized images. This suggests a dilatation of the choroidal vessels.

Histologically, the choroid comprises blood vessels and interstitial tissues. Because the choroid does not have well-organized structures like the retina, it is difficult to detect morphological changes in the EDI-OCT images. Therefore, we used the binarization technique to differentiate and quantify the luminal from the interstitial areas. Sonoda et al. [2] showed that the luminal and stromal areas could be differentiated and quantified with high repeatability and reproducibility. In the present study, a comparison of the original EDI-OCT images to the binary images showed that the traced areas coincided with the dark choroidal areas of the EDI-OCT image. Thus, the binarization technique is not only reproducible and repeatable, but it is also valid. Although we did not have definitive evidence that the dark areas truly represented the vascular areas and the light areas the interstitial tissues, the findings of other reports and numerous empirical observations strongly suggested that the dark areas were the vascular components [5,6].

As in our patient, the large retinal AVMs in Wyburn-Mason syndrome usually cause cystic retinal degeneration [4,7-9]. Onder et al. [10] reported a patient with Wyburn-Mason syndrome who developed serous retinal detachment and cystoid macular edema. The authors suggested that impairments of the choroidal vessels related to Wyburn-Mason syndrome may have led to serous retinal detachment and cystoid macular edema [10].

The retinal AVMs are typically stable and usually do not change [11]. However, there are a few reports of spontaneous changes in the retinal AVMs (e.g., expansion, elongation, dilation, increased tortuosity, and regression) [12,13]. In our case, the vascular loops in some areas became more dilated and tortuous in 16 years.

There are several reports of cases with visual disturbances due to progressive optic neuropathy accompanied by orbitocranial AVMs [11-13]. Reck et al. [11] studied 21 cases with Wyburn-Mason syndrome that were confirmed by imaging or autopsy information. They reported that 11 of the 21 cases presented with symptoms attributable to intracranial AVMs and seven cases with symptoms attributable to optic neuropathy. Progressive visual disturbances over several years due to optic neuropathy may result from a compression of the retrobulbar or intracranial optic nerve by enlarged orbital or retro-orbital AVMs. The optic nerve damage results from orbital congestion and compression of the nerve in the optic canal, where there is relatively little space for expansion of the vasculature. In our case, a similar mechanism may have contributed to the decreased visual acuity.

## Conclusion

This is the first study reporting an increase in the choroidal thickness and the luminal area in an eye with Wyburn-Mason syndrome. Further studies will be needed to determine whether a thickened choroid is characteristic of all cases with Wyburn-Mason syndrome. The conversion of EDI-OCT images to binary images may be a useful method to quantify the choroidal structure.

### Consent

Written informed consent was obtained from the patient for publication of this case report and any accompanying images. A copy of the written consent is available for review by the Editor of this journal.

## Abbreviations

AVM: Arteriovenous malformation
EDI-OCT: Enhanced depth imaging optical coherence tomography
FA: Fluorescein angiography
IA: Indocyanine green angiography
OCT: Optical coherence tomography
SD-OCT: Spectral-domain optical coherence tomography
